# On the Treatment of Fibroids of the Uterus by Electricity

**Published:** 1887-09

**Authors:** W. Windham Webb

**Affiliations:** London


### Selections.

### *ON THE TREATMENT OF FIBROIDS OF THE UTERUS BY ELECTRICITY.*

By W. WINDHAM WEBB, M. D., M. R. C. P., London.

*The Operation.*—Dr. Apostoli does not pretend to make any innovations upon the current theoretical notions as to the treatment of fibroids of the uterus. While others with caustics, tents, scrapings, injections, incisions, ergot, and operations aim at renovating the mucous membrane of the organ, setting up a derivative process, bringing about a dissolution and absorption of the tissues, contraction of the tumor, and diminution of its bulk, with coincident relief of the attendant symptoms, he maintains that his way of gaining the same ends is by means simpler, easier, certain, and more exactly regulated. There is no mysticism about the matter. Others had essayed the electrical treatment, but not to their complete satisfaction. To account for this was a piece of plain reasoning. If such and such an agent were known to be able to produce such effects, and those effects have not been obtained, the natural conclusion was that there must have been some defect in the method of application, some mischoice of the seat, or some miscalculation in the amount of power employed. It was not difficult to make out that such was the case here. More careful study of the local action of electricity, and experience in the way of utilizing it, disclosed the source of failure. It was proved that much stronger currents than was supposed could be directed upon the internal surface of the uterus, that they could be made to pass through the tissues of a tumor effectively, that the circuit could be completed through the skin harmlessly, and that, by means of the galvanometer the intensity could be exactly adjusted to the nature of the case and the tolerance of the patient. Working downwards from principles to facts, practice was at length brought into accord with theory, and the way opened to success. Tumors could not resist electricity boldly and guardedly administrated.

The fact of the two poles having different local effects regulates their use; they both cauterize. The positive pole is coagulating, and it hardens that with which it comes in contact without destroying its vitality. The negative pole, on the contrary, has dissolvent and destructive powers. The coagulating effect of the positive pole is, in the first place, utilized in transforming the hemorrhagic surface of the interior of the uterus into one that will not allow of sanguineous exudation or excessive secretion, and then in causing contraction of the fibro muscular mass, a lessening of its blood supply, a lowering of its nutrition, with a consequent partial atrophy. The negative pole is brought to bear upon the uterine cavity and tumor, when reduction of size by electro-chemical action is the immediate object.

These galvano-caustic operations upon the mucous membrane of the uterus are, therefore, of two kinds, according to the nature of the case, and the pole required to be used. The manipulations are, however, much the same in both. But when, from distortion or displacement, the cavity of the uterus cannot be reached by the sound, another mode of procedure is rendered inevitable. To pass the disintegrating current through the diseased structure, a puncture is made, and this artificial channel may or may not communicate with the cervical canal or the uterine cavity It is either reserved for repeated introductions of the sound carrying the current, or new punctures may be made from time to time, on suitably presenting parts of the tumor.

Supposing that one of the galvano-caustic operations is about to be done, I will detail the separate stages of it in the order of their sequence. After testing the working power of the battery, the integrity of the regulators and conductors, and the exactness of the indications given by the galvanometer, so that there may be no chance of a shock to the patient by an interruption of the current of electricity during its application, the first duty is to insure the perfect purification of the hands and instruments to be employed. To disinfect the sound absolutely, it is better to pass it through the fire, or the flame of a spirit lamp, and when cold to plunge it into carbolized vaselin. The instruments should be arranged in the order they are wanted, within convenient reach of the surgeon, and immersed in a solution of phenol in a shallow tray.

The patient, after proper instructions as to what is about to be done and the part she is to play, is then to be placed in position, laying on her back, with the buttocks projecting over the side of the bed, the feet resting upon chairs, the thighs spread out, the abdomen uncovered, and all the dress unloosed. Chloroform is not often necessary, except when a puncture has to be made. The slab of wet clay, with its conductor partly imbedded in its upper side so as to complete the circuit, is now applied to the lower part of the body, avoiding the thighs and the pubic hairs, after observing that there are no pimples or scratches on the skin. Should any be found, they must be protected with collodion, paper or some bad conductor, so as to guard against the concentration of the current at these spots and the consequent eschars. Putting on the clay at this stage gives time for its close adhesion, and the moistening of the skin. Folded linen or a towel is laid over it, and the hands of the patient served to press it sufficiently.

A free vaginal irritation with the sublimate solution is to be made, followed by a digital examination of the parts. The sound, fixed by a screw into its insulating handle, with so much of its length uncovered by the celluloid sheath as corresponds with the uterine measurement, is next introduced through the cervical canal into the cavity of the organ. This is a part of the operation which demands the greatest care and ability. It must be done without the speculum. The point is made to slip over the pulpy end of the index finger of the second hand, which serves as a guide when placed against the lower lip of the orifice. Every movement in advance or laterally can be thus directed, and assurance can be gained that the sound is fully inserted, and touches the upper end of the cavity. No force must be used, and a gentle insinuating movement will succeed better than a hurried thrust. In retreating from the vagina, the finger should be trusted to gather proof that, in every part of its length, the insulating celluloid sheath, pressing against the neck of the uterus and extending beyond the vulva, is guaranteeing the safety of the canal. Similar precaution about this matter should be continued through the operation, as any slipping of the sheath and baring of the metal would occasion a painful shock, and perhaps derange the position of the sound.

Once satisfied that all is justly arranged and secure, the end of the rheophore coming from the galvanometer is to be made fast in the aperture for it in the handle of the sound, avoiding in the movement any displacement of the instrument, but at the same time making sure that it is not likely to slip out. To give proper support to the sound, and maintain its immobility or direct it, as the point is swept over the mucous membrane of the cavity, it is better to continue the index finger of the hand which holds it in the vagina, or, if it be withdrawn, and the instrument is only kept lightly but firmly between the finger and thumb, the back of the hand should rest against the inner side of the corresponding thigh of the patient.

There must now be a pause of some seconds, so that the woman under operation may recover from any little pain or agitation arising from the introduction of the sound. When this has calmed down, the current of electricity may be turned on, commencing slowly, and not going for the first half minute beyond the strength of [illegible]0 or 30 milliampères. Augment gradually to 70, 80 or 100 milliampères. For the first sitting it is well to be content with this intensity. The dose that can be borne varies with every patient, and at different times with the same patient, but it should be held as a rule never to cause more than an easily supportable degree of pain in any case. Much, however, depends upon the mode of administration. By cautiously raising the amount and following the indications of the facial expression, gaining the woman's confidence by stopping as soon as she shows signs of impatience, and limiting the current by her power of bearing it uncomplainingly, in subsequent sittings an intensity of 150, 200, or, if necessary, 250 milliampères may be arrived at. The maximum once reached, it is continued to the end of the operation without change, unless the patient urges a diminution. The operation may be made to last, according to circumstances, for four, six, eight or ten minutes.

The current must be stopped as it was begun, never abruptly, for that would produce a shock. Even with all precautions, the inverse current which is set up when that which has been used is suspended mostly causes a sensation of renewal, so that the patient fancies you are recommencing. The vaginal electrode is now to be detached, the sound carefully withdrawn, and the clay removed from the abdomen. If all has been properly done, the skin will be found cool and without any redness. A vaginal irrigation follows, and a strip of iodo form gauze is introduced into the vagina. The patient should be kept in bed for some hours, or, at any rate, made to avoid exercise or fatigue, and cohabitation with her husband should be forbidden. Uneasiness or slight pain in the abdomen is not an uncommon experience, and it is well to forewarn the patient of the probability; telling her at the same time that it will go off with rest. She must also be cautioned not to feel surprised should she find, for the first twenty-four hours, a small appearance of blood, and afterwards a little purulent discharge. Change the gauze, and wash out the vagina with the sublimate solution every day till the next operation.

The preliminary proceedings are the same when it is intended to have recourse to the negative galvano-puncture. The trocar is to be disinfected, fixed in the handle, and a sheath of celluloid fitted to it, so as to leave exposed as much of the point as may be wanted for a puncture of one, two or more centimètres. Before being used, the whole should be dipped in strong carbolic solution. As the negative pole is made use of with these punctures, the trocar may be of steel. Platinum is too soft to retain a point sharp enough to penetrate hard tissues. Taking off the sheath, it is passed into the vagina and its distal end pressed against the spot chosen for the puncture. The trocar is then steadily thrust through it and into the substance of the uterus or tumor, while an assistant with his hand above the pubes holds the mass immovable in its place. The rheophore is attached, and the current set in motion. The depth of the puncture, the force and duration of the current, are matters to be decided upon by the operator in taking account of the peculiarities of the case. No formidable punctures are required, and it is advisable to restrict the length of the piercing part of the trocar to the smallest extent that will answer the purpose, always bearing in mind the sometimes abnormal situation of the bladder and the possibility of touching large blood-vessels. The same intensity of current may be employed as for the cauterization of the uterine cavity. The daily vaginal irrigation and change of gauze must be attended to, and the patient should be kept quiet, to avoid suffering and any unpleasant consequences from fatigue or irritation.

The repetition of these operations must be determined by a variety of considerations coming out of the general condition of the patient and the changes going on in the diseased parts. Intervals of two, three or four days may be sufficient when complete repose can be indulged in. With other cases and circumstances, a much longer time may be given. The number of sittings required may be from three or four or more than thirty. Generally the later operations are better supported than the earlier ones, but at no time is the pain more than many patients submit to without murmuring, though where there is any nervousness or apprehension it is prudent to give chloroform to avoid the mischief that might happen from involuntary starting.

There are many practical details and matters of experience, which, when well known and observed, have a great influence upon the success of this method of practice, though separately they may seem to be of comparatively small importance. These would fill up too much space, and must be sought for in the writings of Dr. Apostoli, or, what is much better, by personal observation, by anyone who means to take in hand the operations which we are considering.—*British Medical Journal.*

